# Dietary Inference from Upper and Lower Molar Morphology in Platyrrhine Primates

**DOI:** 10.1371/journal.pone.0118732

**Published:** 2015-03-04

**Authors:** Kari L. Allen, Siobhán B. Cooke, Lauren A. Gonzales, Richard F. Kay

**Affiliations:** 1 Department of Anatomy & Neurobiology, Washington University Medical School, 660 S. Euclid Ave., Box 8108, St. Louis, Missouri 63110, United States of America; 2 Department of Anthropology, Northeastern Illinois University, 5500 N. St. Louis Avenue Chicago, Illinois 60625, United States of America; 3 Department of Evolutionary Anthropology, Duke University, Box 90383, Durham, North Carolina 27708, United States of America; University of Florence, ITALY

## Abstract

The correlation between diet and dental topography is of importance to paleontologists seeking to diagnose ecological adaptations in extinct taxa. Although the subject is well represented in the literature, few studies directly compare methods or evaluate dietary signals conveyed by both upper and lower molars. Here, we address this gap in our knowledge by comparing the efficacy of three measures of functional morphology for classifying an ecologically diverse sample of thirteen medium- to large-bodied platyrrhines by diet category (e.g., folivore, frugivore, hard object feeder). We used Shearing Quotient (SQ), an index derived from linear measurements of molar cutting edges and two indices of crown surface topography, Occlusal Relief (OR) and Relief Index (RFI). Using SQ, OR, and RFI, individuals were then classified by dietary category using Discriminate Function Analysis. Both upper and lower molar variables produce high classification rates in assigning individuals to diet categories, but lower molars are consistently more successful. SQs yield the highest classification rates. RFI and OR generally perform above chance. Upper molar RFI has a success rate below the level of chance. Adding molar length enhances the discriminatory power for all variables. We conclude that upper molar SQs are useful for dietary reconstruction, especially when combined with body size information. Additionally, we find that among our sample of platyrrhines, SQ remains the strongest predictor of diet, while RFI is less useful at signaling dietary differences in absence of body size information. The study demonstrates new ways for inferring the diets of extinct platyrrhine primates when both upper and lower molars are available, or, for taxa known only from upper molars. The techniques are useful in reconstructing diet in stem representatives of anthropoid clade, who share key aspects of molar morphology with extant platyrrhines.

## Introduction

Numerous studies document a general relationship between primate molar occlusal morphology and diet, providing a useful way of inferring the diets of extinct species. To date, the bulk of this literature has focused on the use of lower molars in dietary reconstruction, with little consideration of the corresponding upper molar morphology. Among primates, measurements of the relative length of the lower molar shearing crests [1,2,3] and various measures of occlusal surface relief have been explored as correlates of diet in extant groups [4,5,6,7]. With the exception of a small data set of strepsirrhine upper molar crest lengths explored by Bajpai *et al*. [8], it has not yet been established whether a reliable diet signal may be captured from upper molar occlusal morphology as well. Furthermore, it is debated whether measures of occlusal relief and relative lengths of the shearing crests are comparably reliable indicators of diet category [5]. Here, we present a comparison of dietary signals present in occluding pairs of upper and lower first molars in medium- to large-bodied platyrrhines—a clade of primates for whom the phylogeny is well understood—using multiple measures of occlusal morphology (shear quotients and occlusal surface relief indices). Finding a generalizable method for inferring diets of extinct platyrrhines from upper as well as lower molars would introduce a previously unutilized source of information about platyrrhine paleobiology. Also, because Eocene-Oligocene anthropoids are similar to platyrrhines in body size and molar structure, this data set will provide an added source for inferring early anthropoid niche structure to that based on lower dental morphology [9,10].

In the introduction to their 1984 book “*Adaptations for Foraging in Nonhuman Primates*” Rodman and Cant [11] call attention to three sorts of adaptations: those for reproduction, avoidance of predation, and acquisition of food (foraging). Of the three, they note,

“… it is the ‘hunt’ and capture of food that seems to select most consistently for organismal design in primates…. Physiological potentials determine the suitability of any food to any specific forager, and physical structure of the food determines the potential of the forager to reduce the food to useful form” (page 2).

Consistent with Rodman and Cant’s observation, foraging and dietary specializations play a fundamental role in theories about the origin of the evolutionary novelties that characterize primate, anthropoid, and human origins [2,8,10,12,13,14,15,16,17,18,19]. Not surprisingly, the search for morphological signals of diet in fossils has produced an enormous literature emphasizing morphology, tooth wear, enamel structure, and the isotopic and physical properties of dental materials [20,21,22,23,24,25,26].

Most studies of diet-morphology correspondence emphasize how upper and lower cheek teeth interact to reduce food size during mastication [27,28]. It is assumed that tooth occlusal surfaces are subject to natural selection related to chewing efficiency [29] and to the ability of the teeth to remain functional in the face of chewing forces and attendant wear [30]. Increases in the surface area of masticated food as a consequence of chewing should increase the rate of digestion, thereby increasing the rate of energy return [31,32]. Thus, the structural properties of the food (hardness, ductility, etc.) select for the structural design of the teeth through adaptation for more efficient trituration and maintenance of functionality [33]. At the same time, tooth structure is selected to optimize the extraction of nutrients from food; foods like sugars and starches may not have to be chewed finely to achieve an optimal digestibility, whereas plant or animal structural carbohydrates require extensive oral processing to optimize digestibility [31]. Hard object consumers, for whom food processing and maintenance of dental function may be a balancing act, can often be differentiated from taxa consuming softer items through enamel specializations in combination the very low molar relief (e.g., [23]). Additionally, body size must also be taken into consideration owing to its dual association with metabolic needs and food passage time [31,34,35].

Some work has been done to examine the relationship between dental topography (molar crest lengths), chewing efficiency (chewed food particle size) and food physical properties. Sheine and Kay [29,32] report experiments showing that the relative lengths of molar shearing crests in six strepsirrhine species are related directly to efficiency in increasing food surface area. Yamashita [36] finds that lower molar crest lengths are correlated with leaf consumption in five Madagascar lemur species, such that animals consuming a higher proportion of leaves and materials with higher shear strength (measured as the stress at which the material tears, in kg/mm^2^) tend to have longer lower molar shear crests, relative to lower molar area. Likewise, research by Kay [1,2] has demonstrated that the degree of development of the cutting edges of the lower molars (measured in three dimensions with a binocular microscope, not as projected onto the occlusal plane) when corrected for tooth size and expressed as a Shearing Quotient (SQ) serves as a guide to the diet of extant species. A modification of this approach using Shearing Ratios (SR) used by Strait [3] yields similar results.

Recently, a number of techniques employ laser and μCT scanning to produce three-dimensional models of tooth crowns. A variety of different measures to capture the surface topography can then be collected. Several indices derived from surface topography have been demonstrated to correlate with dietary profile in select mammalian groups. These include measures of occlusal relief (e.g., Occlusal Relief, (OR; [4]), Relief Index (RFI; [7]), Orientation Patch Count (OPC; [37]), and Dirichlet Normal Surface Energy (DNSE; [6]). Relief indices (RFI and OR) represent the ratio of the three-dimensional surface area to the two-dimensional tooth cross-sectional area. The greater the occlusal relief, the higher the values of these indices [38]. Originally developed to study tooth wear, measures of molar relief (RFI and OR) have been applied to the study of surface topography of unworn teeth to search for diet-morphology correlates in euarchontan mammals [7], strepsirrhines [5], platyrrhines [5,39], and hominoids [4]. Unlike measurements of relative molar shear (SQ), no laboratory or field studies have examined whether mechanical food properties and/or food shape are correlated and functionally related to the three-dimensional dental topographic variables.

Simple proxies of tooth function and dental topographic methods each have strengths and weaknesses [6,7,39]. All techniques are sensitive to dental wear as would be expected from simple examination of the gross effects of tooth shape as teeth wear. Therefore, study samples usually include specimens that have little wear. Phylogenetic effects also must be considered in interspecific comparative analyses on a large number of species, to account for the likelihood that a part of the shared similarity between closely related species may be due to shared descent [7]. Inter-individual error in measurement has been considered a further potential drawback of shearing measurements because the technique is based on recognition of landmarks that often are difficult to identify precisely. Equally, however, surface topographic methods are not entirely landmark-free because the observer must choose how to ‘crop’ the tooth crown from the root and determine the type and degree of smoothing algorithms to apply to the surface prior to analysis.

Finally, we recognize an overarching problem of each of these current approaches is quantifying diet as a categorical variable to compare with molar structure. If we are evaluating the statistical ‘success’ of assigning a species to a particular diet category using a particular measure of molar morphology, we need to feel confident that the within-group variability is less than the between-group variability in the diet categories, for example that items within each dietary category have broadly similar structural properties [36]. The state of our current knowledge does not permit this except in a handful of cases.

Bearing in mind the above caveats and uncertainties we analyze occluding upper and lower molar structure in a sample of platyrrhines, with intent to evaluate the feasible use of upper molars in diet reconstruction, and to compare the efficacy of three methods of dietary inference—RFI, OR and SQ. Our dataset consists of thirteen species of medium to large-bodied New World monkeys. The phylogeny of the group is well known (see Materials) allowing us to measure and control for phylogenetic effects in the data. Large samples are used when calculating each index for the associated upper and lower molars of the same individual specimens. Previous evaluations of these measures focused on lower molars, whereas our new dataset includes measurements of both the lower teeth and the upper teeth with which they occlude.

We pose two questions: Controlling for sample size and phylogenetic effects, how efficiently do RFI, OR, and SQ of the lower and upper teeth correctly assign individuals and species to dietary categories? And, how do these measurements used together and in various combinations affect the accuracy of the assignments?

## Materials and Methods

### Data collection

The study sample consists of the upper and lower first molars of thirteen species of platyrrhine primates (an average of 7 specimens per species) covering a range of body sizes and dietary profiles (Table 1). All specimens used in this study were collected by mammalogists and are housed in systematic collections in the United States and Brazil. A full list of the specimens (with museum attribution) is found in S1 Table. No animals were sacrificed for the purposes of this study. Representatives from all three extant platyrrhine families were included; however, members of the subfamily Callitrichinae (marmosets and tamarins) were excluded. Due to the uniformly small size of callitrichines, the scanning tools we employed for surface analyses lack sufficient precision for these species to be accurately measured. Furthermore, early platyrrhine and catarrhine fossils—the target species for dietary reconstruction by these methods—demonstrate body sizes more consistent with extant non-callitrichine platyrrhines [19,40,41,42,43,44]. The sample was restricted to individuals with no more than minimal tooth wear. As a wear criterion, a specimen was rejected if the enamel was perforated to the dentine beyond the cusp tips (a simple circular exposure of dentine at cusp tips was acceptable but a ribbon of dentine exposure along a crest led to rejection). Specimens were also rejected when postmortem defects and breakage were encountered. Dietary information reported here is based on mean annual diet taken from the literature (see description in Analysis section) [45]. We calculated SQ and RFI for both the upper and lower first molars of each individual. These two dental measurements have previously been applied to lower molars and demonstrated to be useful for differentiating primates by diet. A third measure, (OR), was taken only for the lower first molar but not the first upper molar for reasons described in the methods section. Measurements of SQ were taken on high-precision epoxy casts while RFI and OR were calculated from laser scan models of the same casts.

**Table 1:** Platyrrhine sample with dietary profile.

Species	N	Body Mass(grams)	Primary Dietary Components	Diet Category	Diet References
*Alouatta palliata*	7	6250	Leaves, fruit	Folivore	[46,47]
*Alouatta seniculus*	5	5950	Leaves, fruit	Folivore	[48,49,50]
*Aotus vociferans*	9	703	Fruit	Frugivore	[51,52]
*Ateles geoffroyi*	9	7535	Fruit	Frugivore	[53,54,55,56]
*Brachyteles arachnoides*	9	8840	Leaves, fruit	Folivore	[57,58,59]
*Cacajao calvus*	10	3165	Hard objects, fruit	Hard object feeder	[60]
*Callicebus caligatus*	2	880	Fruit	Frugivore	[61,62]
*Callicebus cupreus*	8	1070	Fruit	Frugivore	[61,62]
*Cebus capucinus*	9	3160	Fruit	Frugivore	[53,54]
*Chiropotes santanas*	8	2740	Hard objects, fruit	Hard object feeder	[63,64,65,66]
*Lagothrix lagotricha*	9	7150	Fruit	Frugivore	[62,67,68,69,70]
*Pithecia irrorata*	9	2160	Hard objects, fruit	Hard object feeder	[71]
*Saimiri boliviensis*	9	811	Fruit, insects	Frugivore	Genus-level information: [62,72,73]

See Allen and Kay [46] for detailed dietary information; body mass (in grams) is the average of males and females, from Smith and Jungers [74].

### Shearing Quotient (SQ)

**Fig 1:**
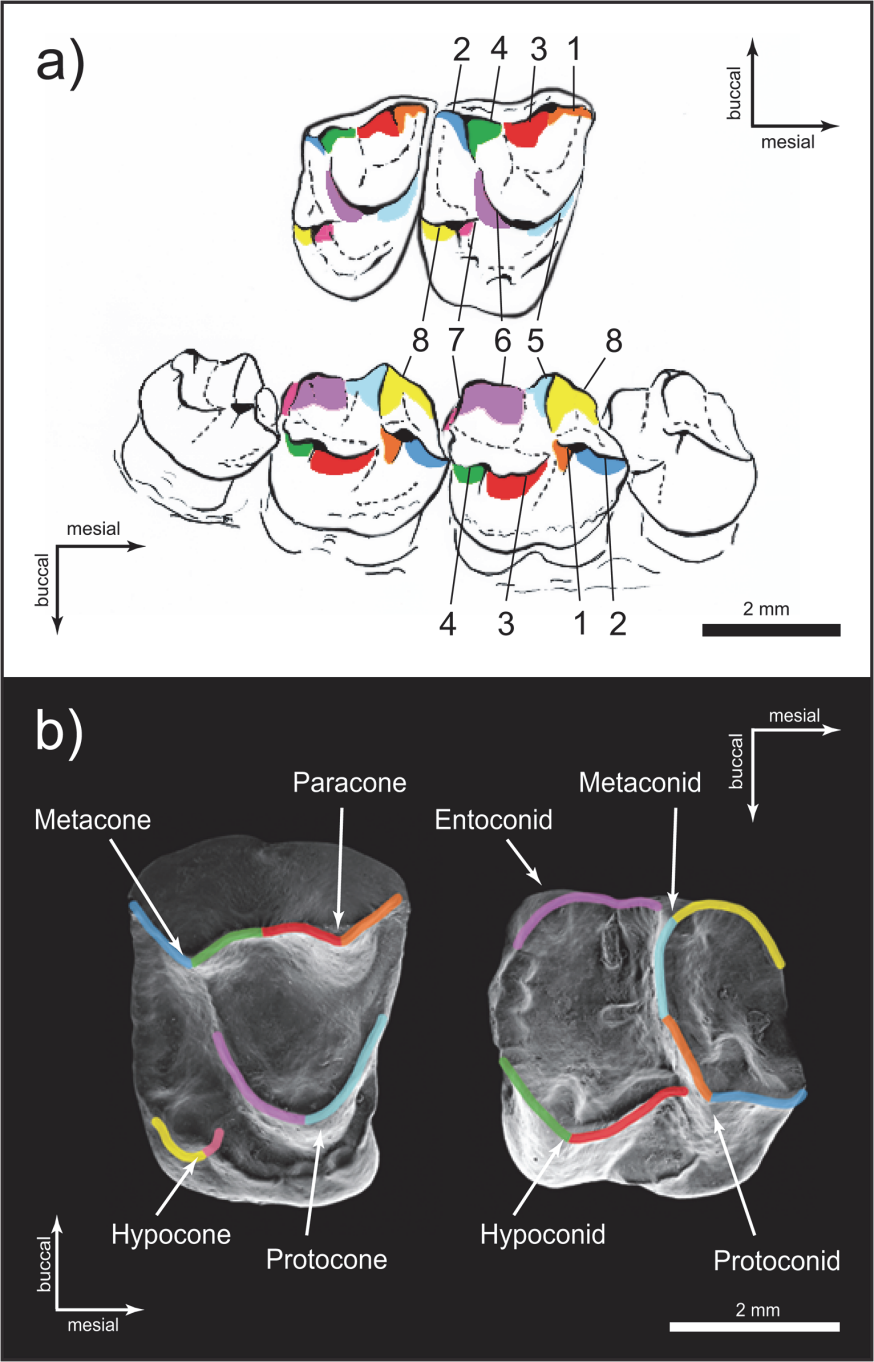
) SEM image of the occlusal surface of the right first maxillary (on the left) and first mandibular (on the right) molars of *Saimiri sciureus* (USNM 546762, Para, Brazil). a Crests 1–6 are shown. b) *Saimiri sciureus* (USNM 546762), oblique medial view (above) of right M^1–2^ and oblique lateral view (below) of P^4^-M^3^. The leading edges of eight functional crests are labeled, as are the wear surfaces distal to them. We measured crest lengths 1–6; crests 7 and 8 (associated with the margins of the hypocone and trigonid basin) were not measured. Numbering system of the crests follows Kay [102].

The lengths of shearing crests 1 through 6 and mesiodistal tooth length were measured for both upper and lower first molars (Fig. 1A,B) under a binocular microscope fitted with a reticle eyepiece at 12x or 25x magnification (depending on the size of the tooth). All measurements were taken in reticle units and converted to millimeters. ‘Total shear’ was calculated as the sum of shear crest lengths 1 through 6. Crest measurements were repeated by three observers (KLA, LAG, and RFK). Inter-observer error in the sum of shearing crests and tooth length was less than 5%.

### Three-dimensional dental relief: Relief Index (RFI) and Occlusal Relief (OR)

Entire maxillary and mandibular post-canine tooth rows were molded, cast, and laser surface scanned following a protocol outlined in Cooke [39].

**Fig 2:**
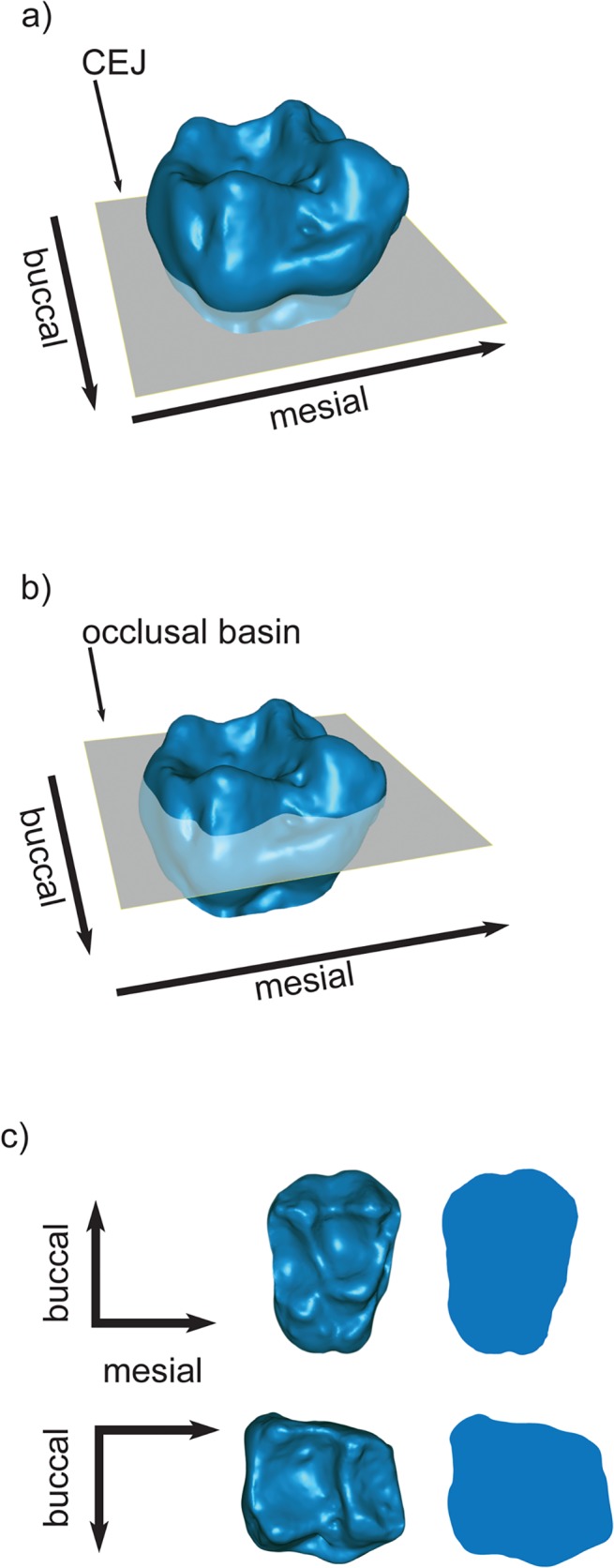
Laser-scan generated image of *Saimiri boliviensis* (AMNH 255858) first molars demonstrating calculations for surface relief measures. See text for calculations. a) and b) oblique lateral view of lower first molar showing cropping at the talonid basin (a) and cemento-enamel junction (b, CEJ); c) upper (top row) and lower (bottom row) first molar in occlusal view with planometric surface area projection depicted to the right.

Relief Index (RFI), a measure of the overall relief of a tooth crown, was calculated following Boyer [7] as ln(√TSA/√PSA) where TSA is the total surface area of the enamel crown cropped along the cemento-enamel junction (CEJ) and PSA (projected surface area) is the two-dimensional surface of the projection of the outline of the molar oriented in the occlusal view (Fig. 2). This procedure was completed for both upper and lower first molars. To examine the degree of intra-observer error that occurred during the cropping procedure one molar specimen was cropped ten times on several different days. Error was found to be less than 2% (S2 Table).

Occlusal Relief (OR) [4], was calculated as a ratio in which the numerator is the three-dimensional surface area of the occlusal surface tooth cropped from the lowest point in the talonid basin, and the denominator is the two dimensional planometric surface area of the occlusal table (Fig. 2). To determine the lowest point in the talonid basin, we passed a plane through the tooth parallel to the occlusal plane (x, z plane) using the “intersect a plane” function in *Geomagic Studio* 2012 (Geomagic, Inc.). The plane was lowered until it intersected only with the lowest point in the basin. The tooth was then cropped below this plane. OR was developed by M’Kirera and Ungar [4] for use on lower molars. The upper molars in our sample displayed considerable variation in cingulum development, which created difficulty in establishing a consistent cropping procedure. As such, we limited the use of OR in this study to the lower molars. RFI and OR were measured and calculated by SBC.

For both the upper and lower molars, Log10 TSA scales isometrically with Log10 PSA (i.e., the slope does not significantly differ from 1.0), so we followed the previous literature [4,7] in representing both as ratios rather than residuals. Lower molar TSA scales isometrically with molar length (slope = 2.0), whereas the upper molar TSAs scale with slight negative isometry (slope = 1.78, 95% CI for slope = 1.72 to 1.84).

### Analysis

Closely related species may be expected to be more similar morphologically, owing to a greater degree of shared evolutionary history. A high degree of phylogenetic signal, or covariance between the species data and their phylogenetic relationships, may violate the assumption of independence of data points inherent in traditional correlation analyses [75,76]. We tested for the effects of phylogenetic signal in our variables by calculating Pagel’s lambda (λ) in the ‘geiger’ package for the statistical program ‘R’ [77]. Three fully resolved platyrrhine phylogenies with associated branch lengths were used, representing variable arrangements at the subfamily level: the maximum parsimony and maximum likelihood trees from Opazo [78] and the platyrrhine phylogeny from Perelman *et al*. [79].

Because the sum of the shear crests does not scale isometrically with molar length, the use of a ratio (sum shear/tooth length) fails to properly account for body size effects. Instead, SQs were calculated as the residual sum of the shear lengths using mesiodistal tooth length as a proxy for size. SQs are calculated in the following way: Using the ‘caper’ package for ‘R’ [80], a phylogenetically-corrected least-squares regression (PGLS) line was fitted to the Log10 species means of upper and lower first molar lengths (on the abscissa) and Log10 summed first molar shear (on the ordinate) of species means. The derived linear equation was used to generate an ‘expected’ shearing crest length from molar length of each included individual or taxon. First, expected and observed variables were converted to real space. The expected value was then subtracted from the observed value for each taxon, and divided by the expected value. The resultant (SQ value) is expressed here as a percentage.

**Fig 3:**
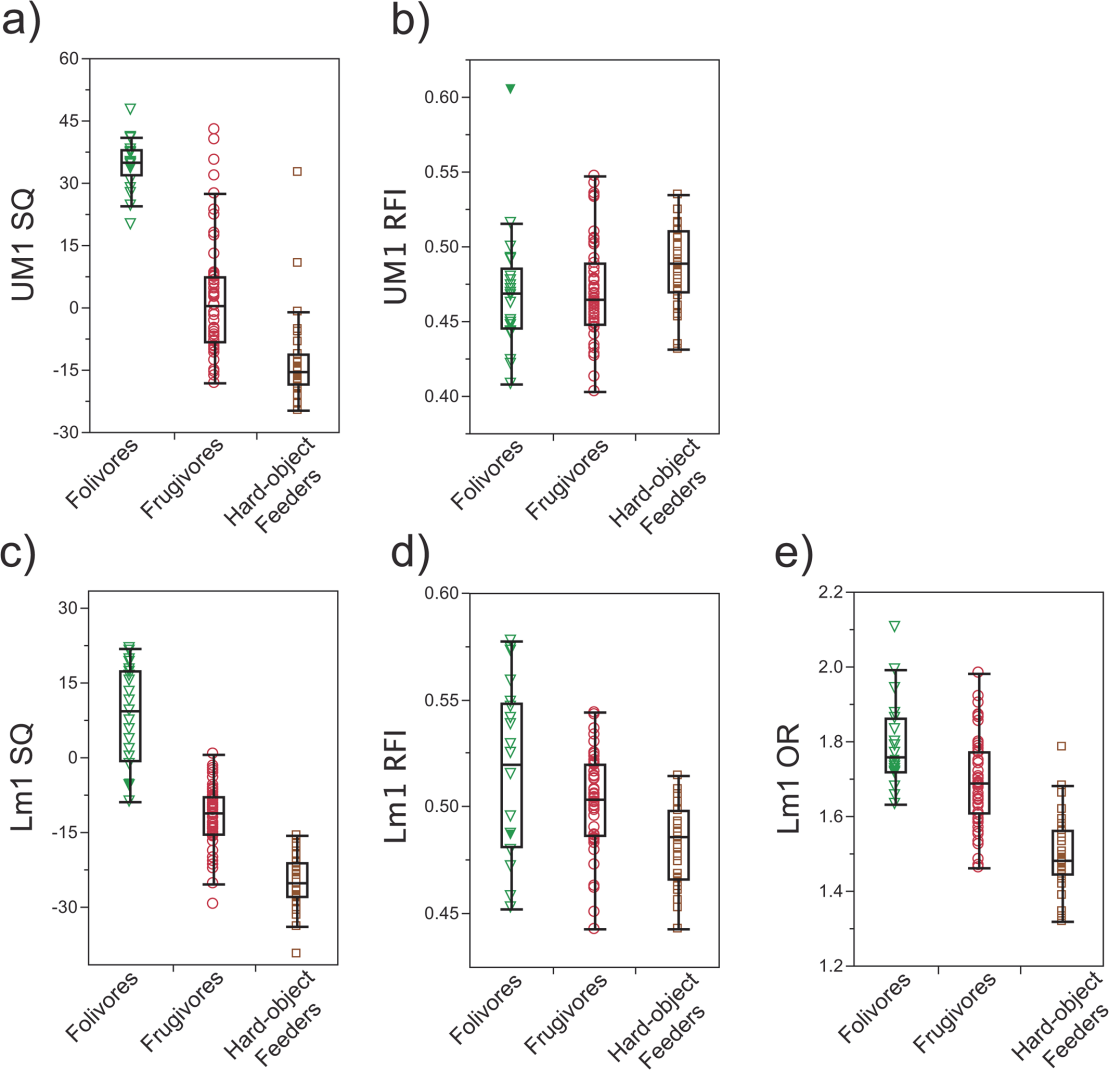
Box-and-whisker plots showing ranges of individual values for Diet-1 (3 group comparisons). a) upper molar SQ, b) upper molar RFI, c) lower molar SQ, d) lower molar RFI, e) lower molar OR.

Allen and Kay [46], synthesize the available behavioral data concerning platyrrhine diet composition, based on recorded feeding times and/or number of feeding bouts per food type [45]. In our analyses, taxa were categorized into feeding groups—frugivore, folivore, and hard object feeder—based on these feeding records. Species for which the mean annual percentage of the recorded feeding bouts/feeding time devoted to consuming leaves exceeded 50% were designated as “folivores”. Species in which fruit is the primary ingested component are categorized as “frugivores”. Species described in the literature as consuming a substantial amount of hard seeds and nuts in their diet are categorized as “hard object feeders”. Table 1 summarizes the primary diet components and assigned diet categories of each species. The SQ regression slopes described above were fit to frugivorous taxa alone. To visualize the distribution of values across different taxa and different dietary groups, SQ, RFI, and OR were plotted in standard box-and-whiskers plots in *JMP Pro* 10.0.2 for Mac, SAS Institute Inc. (Fig. 3A-E), and Kruskal-Wallis tests were computed to look for differences among species and diet categories. Because SQ values (residuals from the PGLS line) were found to contain a phylogenetic signal (λ = 1.0), a phylogenetic ANOVA was computed to test for differences among diet groups using the “phytools” package in “R” [81]. OR and RFI lambda values do not significantly differ (p>0.05) from 0 (OR: λ = 0.4, M^1^ RFI: λ = 0, M_1_ RFI: λ = 0), so traditional non-parametric statistical techniques were used. (The power to detect phylogenetic signal drops dramatically at small sample sizes (*n <* 20 species) [82], so there may, in fact be an undetected phylogenetic signal, a possibility that should be tested whenever samples are sufficiently large.) We applied *post-hoc* multiple pair-wise comparisons (Wilcoxon Rank Sum) to test for significant differences in SQ, RFI, and OR among diet categories. Significance criteria were determined using a Bonferroni correction [83].

We employ discriminant function analyses (DFA) to examine the success rate of classifying individual platyrrhine specimens by diet from upper and lower first molar SQ and RFI, and lower molar OR. These analyses were conducted in *SPSS* Version 21.0 (SPSS, Inc.). M_1_ length and M^1^ length were included as proxies for size. DFAs were conducted by entering independent variables together with all prior probabilities equal to avoid sampling bias as a result of unequal number of individuals assigned to each dietary category. Results were cross validated using “leave one out classification”. Cross-validated classification rates are reported here. Additionally, the data are partitioned so that the classification success rates using all variables can be examined. The relationship among individual data points and group means are visualized in a plot of scores in canonical space, illustrating the axis of variation that provides the best differentiation among group means.

## Results

Table 2 presents species means and standard deviations for all dental indexes. Box plots demonstrating distributions of molar indices by diet group are presented in Fig. 3.

**Table 2:** Species means and standard deviations (SD) for dental indexes.

	UM1 OR		UM1 RFI		Lm1 RFI		UM1 SQ		Lm1 SQ	
Species	Mean	SD	Mean	SD	Mean	SD	Mean	SD	Mean	SD
*Alouatta palliata*	1.72	0.06	0.46	0.07	0.5	0.02	35.72	2.1	12.54	8.6
*Alouatta seniculus*	1.82	0.12	0.47	0.03	0.55	0.04	34.71	6.19	25.87	7.73
*Aotus vociferans*	1.73	0.09	0.45	0.03	0.54	0.02	0.18	6.67	5.11	4.07
*Ateles geoffroyi*	1.7	0.07	0.52	0.02	0.53	0.02	18.46	12.24	0.17	3.25
*Brachyteles arachnoides*	1.84	0.13	0.48	0.02	0.57	0.02	33.52	8.04	22.83	8.65
*Cacajao calvus*	1.51	0.1	0.5	0.02	0.51	0.02	-5.75	16.34	-12.01	5.43
*Callicebus discolor*	1.61	0.06	0.45	0.03	0.54	0.02	-9.25	4.7	0.307	2.6
*Callicebus moloch*	1.65	0.04	0.46	0.02	0.53	0.01	-6.95	4.26	-3.04	4.79
*Cebus capucinus*	1.59	0.11	0.45	0.03	0.51	0.03	-11.74	3.98	-6.86	6.82
*Chiropotes satanas*	1.46	0.08	0.48	0.02	0.49	0.01	-15.4	7.37	-13.8	4.3
*Lagothrix lagotricha*	1.69	0.1	0.47	0.02	0.53	0.02	11.45	19.12	4.53	4.7
*Pithecia irrorata*	1.53	0.13	0.48	0.03	0.51	0.02	-16.67	3.28	-14.15	6.6
*Saimiri boliviensis*	1.85	0.09	0.47	0.01	0.53	0.02	4.82	6.28	-1.22	-5.68

Sample sizes are listed in Table 1.

### Shearing Quotient (SQ)

Results for SQ equations and residual distributions are consistent across all three phylogenies, indicating that the results are robust to deviations in family-level branching patterns. In the absence of differences we present only the results for the Perelman *et al*. [79] tree, which depicts Pitheciidae as the basal crown platyrrhine clade and *Aotus* as the first branch from the Cebidae. The family level branching patterns for this phylogeny are in concordance with data provided by *Alu* insertions, which are considered to have very low rates of homoplasy [84]. Among frugivores in our sample, the sum of the shear crests scale with negative allometry on tooth length for both the upper molars (log10 sum of M^1^ shearing crests = 0.44614 + 0.73751 * log10 M^1^ length; R-square = 0.72, p = 0.0006) and lower molars (log10 sum of M_1_ shearing crests = 0.29713 + 0.91435 * log10 M_1_ length; R-square = 0.97, p = 0.0003) in the PGLS regressions.

Frugivorous platyrrhine species are distributed around the grand mean for the total dataset, as expected from the method by which SQ is calculated (Table 2, Fig. 3A, C). For both the upper and lower first molars, SQ values for folivores occupy the upper end of the platyrrhine range, indicating a higher sum of shear crest lengths relative to tooth length than in frugivores, while hard object feeders occupy the low end of the sample distribution, indicating less molar shearing than in frugivores. The distributions of the upper molar SQ overlap considerably among the three diet categories, largely owing to the broad spread of values within the frugivore group. In particular, *Ateles*, has the highest M^1^ SQ values for the frugivores in our sample. Lower molar SQ values provide a greater degree of separation among diet groups owing to a more restricted frugivore range, although some specimens of *Ateles* still fall within the folivore range. For the upper and lower molars, folivore and hard object feeding categories occupy entirely separate ranges, with the exception of two individuals of *Cacajao*, whose upper molar SQ overlaps with frugivore SQs.

Distributions of indices by diet are summarized in Table 3. A phylogenetic ANOVA on species means indicates significant differences among groups (M^1^: p = 0.005, M_1_: p < 0.001). *Post-hoc* pair-wise comparisons on individual data points indicate highly significant differences among all diet categories (p = <0.0001; Bonferroni significance criterion: p ≤ 0.016) for both upper and lower molar SQ (Table 4).

**Table 3:** Sample parameters for M^1^ and M_1_ occlusal variables grouped by diet.

Diet Category	M^1^ OR			M^1^ RFI			M_1_ RFI			M^1^ SQ			M_1_ SQ		
	Mean	95% Conf. Int.		Mean	95% Conf. Int.		Mean	95% Conf. Int.		Mean	95% Conf. Int.		Mean	95% Conf. Int.	
leaves	1.8	1.85	1.74	0.47	0.49	0.45	0.54	0.56	0.52	34.54	37.26	31.81	20.04	24.58	15.67
fruit	1.7	1.73	1.67	0.47	0.48	0.46	0.53	0.53	0.52	2.27	6.17	-1.62	0.09	1.75	-1.56
hard objects	1.5	1.54	1.46	0.49	0.5	0.48	0.51	0.52	0.5	-12.25	-7.63	-16.87	-13.25	-11.104	-15.57

Values for 95% Conf. Int. indicate the upper and lower bounds of the 95% confidence interval for the diet grouping. See text for additional abbreviations.

**Table 4:** Results of post-hoc multiple pair-wise comparisons between tooth indices (Wilcoxon each pair) segregated by diet categories.

Diet Category		p-value Wilcoxon each pair									
Group 1	Group 2	M_1_ OR		M^1^ RFI		M_1_ RFI		M^1^ SQ		M_1_ SQ	
frugivore	folivore	0.0034	*	0.8235		0.2453		<0.0001	*	<0.0001	*
hard object	folivore	<0.0001	*	0.0126	*	0.0079	*	<0.0001	*	<0.0001	*
hard object	frugivore	<0.0001	*	0.007	*	<0.0001	*	<0.0001	*	<0.0001	*

* denotes statistical significance of p-value using Bonferroni significance criterion for 3 groups (p< 0.0167).

### Relief Index (RFI)

Compared to SQ, both upper and lower molar RFIs show greater overlap of species means among the different dietary groups (species data in Table 2, group data in Table 3; Fig. 3B, D). Although means differ among groups, the ranges of individual specimens within each group overlap substantially. Upper molar RFI group ranges overlap completely. Statistically significant differences exist between the hard object feeders and both the frugivore and folivore distributions but not between frugivores and folivores (Kruskal-Wallis pairwise test with a Bonferroni correction criterion (p<0.017).

For lower molars, the folivore mean RFI and maximum individual values exceed those for frugivores, while hard object feeders have the lowest mean and maximum values (Table 3). All three groups have similar minimum values for each diet category (folivore = 0.48, frugivore = 0.47, hard object = 0.47). A Kruskal-Wallis test shows significant differences in hard object feeders versus frugivores and folivores but not between frugivores and folivores (Table 4).

### Occlusal Relief (OR, lower molars only)

The results for M_1_ OR (Tables 3 and 4) mirror those of the lower molar SQs, with significant differences among means for diet groups (Kruskal-Wallis test: p < 0.0001, *post-hoc* pair-wise comparisons: p < 0.0167 for all groups) (Fig. 3E). Folivores have the highest OR values, hard object feeders have the lowest values, and frugivores are clustered around the grand mean of the total dataset. Despite the significant difference in means between frugivores and folivores, these groups show considerable overlap in distribution of individual values.

### Discriminant function analyses

Summary of the results of the DFA analyses are given in Table 5 and S2 Table. Initially each variable was treated separately to determine its success rate in classifying individual specimens. Lower molar SQ is most effective with 82.5% of individuals correctly classified by dietary category, followed by lower molar length (60.2%), lower molar OR (57.3%), and lower molar RFI (44.7%). For all of the variables, classification rates increase substantially with the inclusion of body size information (M_1_ mesio-distal length). Highest classification rates occur when all lower molar variables—including molar length—are considered together (89.3%).

**Table 5:** Summary of correct classification rates for discriminant function analyses using upper molar (M^1^), lower molar (M_1_), and upper and lower molar occlusal variables combined.

Variables	M^1^	M_1_	M^1^ + M_1_
molar length	60.2	60.2	
SQ	68.9	82.5	88.3
SQ + molar length	69.9	87.4	90.3
OR		57.3	
OR + molar length		79.6	
RFI	47.6	44.7	51.5
RFI + molar length	69.9	69.9	81.6
All variables	82.6	89.3	93.2

Values represent percentage of correctly classified specimens.

Of the dietary groups, frugivores had the lowest classification rates. For lower molar SQ, errors occur predominantly with *Cebus capucinus* specimens classified as hard object feeders. In fact, if species group means are used, only *Cebus capucinus* is misclassified. This is unsurprising as some species of this genus do consume hard objects (e.g., *C. apella)*, though *C. capucinus* does not make these food types a major component of its diet. Additionally, two *Ateles geoffroyi* and two *Lagothrix lagotricha* are misclassified as folivores. For the two indices of topography, errors are less patterned and in the case of RFI, correct classification rates are only slightly higher than random (in a classification scheme with three categories, an individual would have a random chance of 33.3% of being correctly classified vs. our classification rate of 44.7%).

Upper molar analyses largely mirror those of lower molars, but have lower classification rates. SQ classifies 68.9% of individuals correctly by diet and RFI classifies 47.6% correctly. The addition of upper molar length improves both rates to 69.9%; when SQ, RFI, and molar length are used together the classification rate is 82.5%. For upper molar SQ, major errors in classification again occur most frequently within the frugivores. All specimens of *C. capucinus* and all but one specimen of *Callicebus cupreus* are classified as hard object feeders. *A. geoffroyi* and *L. lagotricha* individuals are both frequently misclassified as folivores. While these errors mirror results for lower molars they occur more frequently.

**Fig 4:**
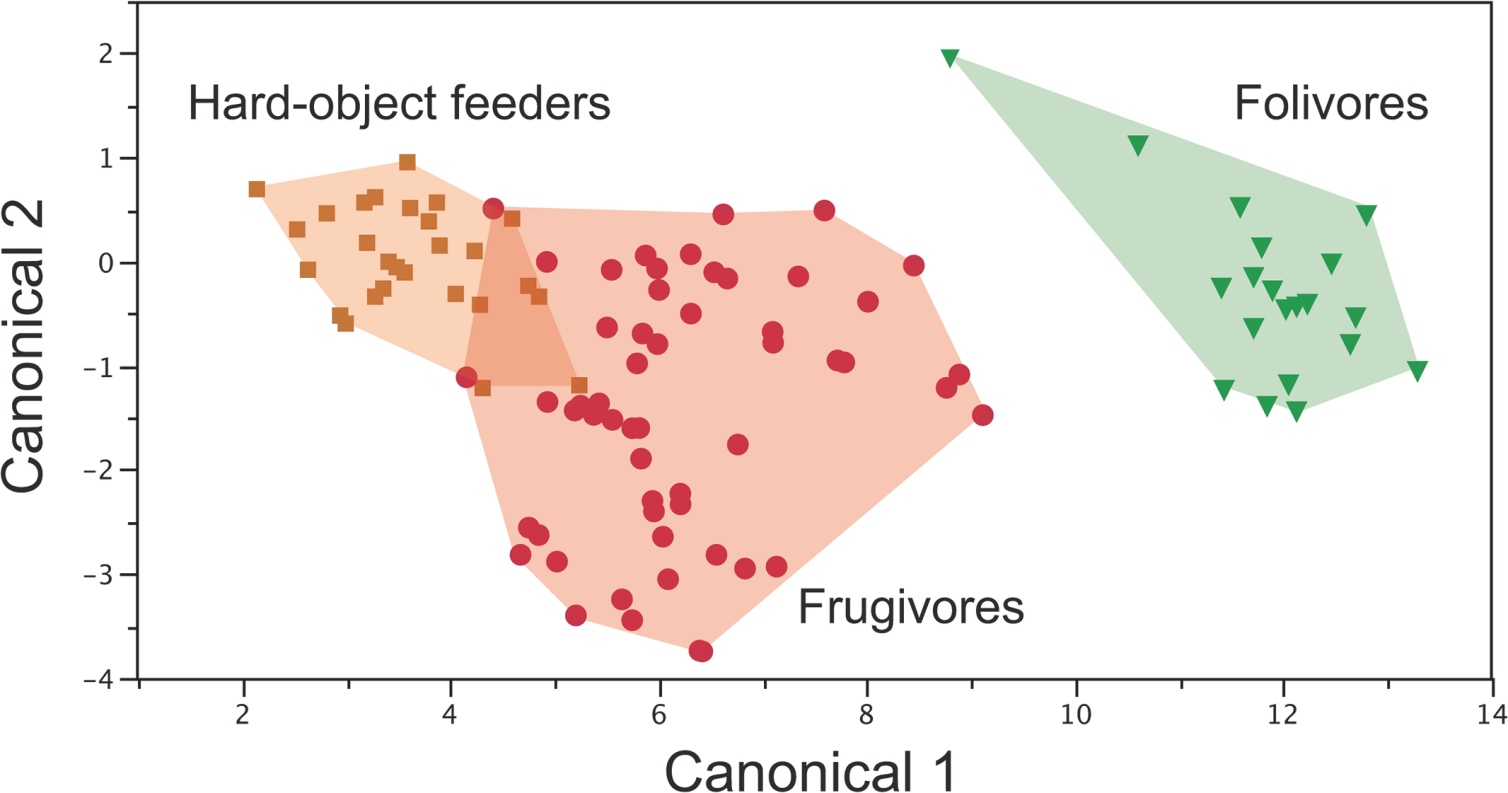
Plot of a discriminant function analysis including M_1_ length, RFI, OR, and phylogenetically corrected SQ and M^1^ RFI and phylogenetically corrected SQ. 93.6% of variance is accounted for by discriminant function 1 and 6.4% by function 2. Polygons are drawn to include all individual specimens.

Classification rates are the highest when all upper and lower molar variables are considered together (93.2%) (Fig. 4). Three of 54 frugivorous individuals are misclassified as hard object feeders and one specimen of hard object feeder (of 27) as a frugivore; of the twenty specimens of folivores, one *Alouatta palliata* is misclassified as a frugivore. In our sample, the combination of upper and lower SQ with molar length returns a classification rate of 90.3%. Some *L. lagotricha* individuals classify as folivores, and a few *C. capucinus* specimens misclassify as hard object feeders.

Upper and lower molar RFI together with molar lengths return a classification rate of 81.6% with major errors occurring when several different individual frugivore specimens were misclassified as hard object feeders. This occurs most often for *A. geoffroyi* and *Saimiri boliviensis*. Without the inclusion of molar lengths, classification rates remain high using SQs of upper and lower molars (88.3%), but classification rates using RFI alone decrease to 51.5%. Indeed, comparison of RFI success rates on upper and lower molars with or without molar length shows that the correct classification rates for RFI and molar length are largely being driven by molar length. Ultimately, as long as molar length is included as one of the variables, most combinations of upper and lower molar variables achieve high rates of classification.

Using species means rather than individual specimens, the three lower tooth indices taken together correctly classify all but one of our 13 species to a diet group (93%), the single exception being frugivorous *Cebus capucinus*, classified as a hard object feeder. The same overall classification rate is achieved using SQ alone (93%), whereas, the success rate for OR alone misclassifies four of 13 species with most of the errors being misclassification of frugivores as either folivores or hard object feeders. RFI performs less well than either of the other lower molar indices, missing 5 of 13 (almost 40%).

Again, when using species means rather than individual specimens, the two upper tooth indices taken together correctly classify all 13 species (100%). Using upper SQ alone, the success rate is 85%, with two frugivorous taxa misclassified as hard object feeders: *Cebus capucinus* and *Callicebus cupreus*. The success rate for upper molar RFI performs less well than either of the other lower molar indices, successfully assigning 10 of 13 taxa (76.2%).

## Discussion

Understanding the relationship between dental morphology and behavior in extant primate species has been a key component for reconstructing the dietary niche of extinct species. Our study examines the occlusal morphology of platyrrhine upper and lower molars, separately and together, in relation to diet, controlling for phylogenetic effects. Several indices designed to capture occlusal morphology (SQ, RFI, and OR) are applied to upper and lower molars of the same individuals among taxa in a restricted taxonomic clade with a highly resolved phylogeny.

Early efforts to infer diet from molar morphology concentrated on allocation of an extinct species into one of several dietary categories [1,16]. But if only one or two specimens of the extinct species are measurable, we cannot establish that the individual values reasonably approximate species means. In such a case, the most conservative approach is to compare the single value with the ranges of extant values. Here, we use DFA to assess the success rate for assigning a single specimen to a particular diet group [5,6,7,39] and to look at success rates for species means of the indices.

An important outcome of this study is the demonstrated significance of using both upper and lower molar data to ‘predict’ dietary behavior in extant species. When training the predictive model using all variables for upper and lower molar concurrently, the predictive power exceeds 90%, even for individual specimens. When possible, we recommend a combination of both upper and lower molar measurements for ‘retrodicting’ the behavior of extinct platyrrhine species. However, when only the upper or the lower first molar is available, predictive success rates remain at ∼85% for individuals and even higher for samples that are sufficient to calculate the species mean with reasonable accuracy. Moreover, the ‘success rate’ exceeds 85% using linear measurements alone. Thus, the more expensive and technologically labor-intensive surface methods do not provide greater accuracy than the simple linear methods.

The findings of this study open the way for more precise interpretation of early platyrrhine evolution. The list of extinct platyrrhine taxa that have both lower and upper molars available, but for which only lower molars have been used in dietary assements, includes Late Oligocene *Branisella* [85,86], Early Miocene *Dolichocebus* [87,88], *Mazzonicebus* [89], *Carlocebus, Soriacebus*, and middle Miocene *Lagonimico* [90], *Cebupithecia, Neosaimiri* [91], *Stirtonia* [92], and *Mohanamico* [93,94]. Added to this are taxa for which there are only upper teeth and consequently, have been excluded from efforts to make dietary reconstructions: Early Miocene *Chilicebus* [40] and “*Kilikaike*” [95].

### Upper versus lower molar dietary signal

Considered either as individual specimens or as species means, indices of lower molar occlusal morphology generally outperform the same measures from the associated upper molars in assigning species and individuals to a commonly used scheme of diet categories. The lower success rate of the upper molar in diet discrimination is largely due to the greater variability of upper molar indices, compared to those of the associated lower molars. Upper molar shear quotient and topographic variables show greater overlap in values among diet categories than do those for the lower molars, resulting in a somewhat greater degree of uncertainty in diet reconstruction. For example, the coefficients of variation for the upper molar SQs are roughly twice those of the lower molars for the frugivore and hard object feeding categories (SI: DFA classification errors). This variation may indicate a relaxed constraint on upper molar shearing in these species, and/or reflect a great deal of variability in the secondary source of nutrition for these species. For example, the frugivorous *Cebus capucinus* may include hard objects (seeds, nuts) in its diet, while *Ateles* supplements its frugivory with young leaves [45].

It is also possible that the greater variability in upper molar topography may reflect phylogenetic effects. For instance, the frugivorous *Ateles* has a lower molar shear quotient in the middle of the frugivore range, but a high upper molar shear quotient more in line with that of its close atelid relatives, the more folivorous *Brachyteles* and *Alouatta*. Nevertheless, upper molar shear quotients do show significant differences among all diet pairings, and a discriminant function analysis of individual specimens for all upper molar measures returns high classification rates. These results indicate that upper molar occlusal morphology is useful for dietary reconstruction, especially when combined with size information (i.e., molar length).

### Topographic versus shear crest measures

Topographic surface measurements of molar relief (e.g., RFI, OR) have been argued to be a three-dimensional version of shearing quotient [38], however, our results show that the incorporation of additional information about surface anatomy does not necessarily replicate SQ results, nor does it dramatically improve the predictive signal for dietary reconstruction. In our study sample, RFI underperformed both SQ and OR for lower molars and SQ for upper molars. RFI was initially designed to quantify morphological differences among dental relief of markedly different morphologies—primates versus other eurachontans [7]. While it has proved useful for that purpose, RFI is not successful here in capturing minor variations in functionally relevant occlusal relief (e.g., shear) within this more phylogenetically circumscribed dataset. Several reasons may account for this discrepancy. RFI describes the relative occlusal surface area of the tooth, a measurement that will be affected by several factors in addition to molar shear, including hypsodonty [5], the presence of accessory cusps and associated crests, as well as other non-occulsal parts of the tooth—sidewall curvature, increasing molar breadth as one nears the CEJ [96], and the presence or absence of a cingulum [39]. Many of these features could in themselves be understood as functionally relevant, compensating for the mechanical demands of food processing, such as resisting dental attrition, or arresting crack propagation. However, feature distribution related to dietary or even non-dietary factors (grit ingested with food, for example) is as yet unclear and may cloud the signal captured by traditional two-dimensional shear measurements.

Many aspects of molar tooth structure picked up by topographic methods may be unrelated to chewing efficiency, as defined by how finely a food is chewed before swallowing with attendant benefits for digestibility. For example, the presence of cingula (low shelves on the crowns of many primate teeth) may be protective, deflecting food particles away from the gingiva [97], or they may reinforce the crown enamel against crack propagation and structural failure [22]. In either event, cingula are not functionally important for trituration, at least until wear is extreme. Another signal that would be picked up by dental topographic studies is enamel crenulation. The crenulated occlusal surface of *Pongo* or *Chiropotes* molars may be a functional design for the routine consumption of relatively tough and hard foods [23,98,99], but relates more to resistance against crack propagation than to masticatory efficiency. Thus, many surface features picked up by dental topographic analyses may well be unrelated to the primary function of reduction of food particle size before swallowing, and in some cases may be correlated effects of enamel structure or thickness. Although these features may convey information on dietary evolution, surface relief measures such as RFI and OPC fail to distinguish surface features like enamel crenulation or cingula from those provided by shearing blades used in trituration. As a result, a surface relief index of a flat tooth containing many crenulations may resemble that of a smoothly curved tooth with multiple shearing blades. Reducing topographic complexity down to a single index would in such a case fail to distinguish two very different adaptive strategies for coping with mastication of food particles requiring fundamentally dissimilar mechanical demands.

In this study, OR yields a somewhat clearer signal than RFI, possibly owing to the elimination of some of the variability in sidewall morphologies, as the tooth is cropped at the lowest point in the basin rather than at the CEJ. This cropping procedure, while feasible and perhaps more appropriate for studies of platyrrhines, is not always possible across a broad range of taxa given the very deep basins seen in some species [7]. Additionally, this method was developed for lower molars; given the greater morphological variability in upper molars, developing a repeatable cropping procedure for these teeth has eluded us. For example, in *Saimiri*, cropping at the low portion of the basin may include the some cingulum in some specimens and not in others.

A recent study by Winchester *et al*. [5] analyzed lower molar shape of platyrrhines. For each individual specimen they calculated a set of relief indices (e. g., RFI, OPC [37]) and linear variables equivalent to SQ. Winchester *et al*. reported a higher classification success rate using dental relief indices than using SQs for discrimination among most of their broad dietary categories. Unlike in many previous studies and the data for lower molars reported here, their version of SQ, which used M_2_ and sampled different but overlapping set of crests, failed to separate platyrrhine folivores from frugivores. In a recently published commentary, Boyer et al. [100] re-analyzed the shearing crest data of Winchester et al., concluding that that the most salient reason for differences between their results and those of prior studies (and also this study) are attributable principally to the tooth position examined: they used the lower second molar whereas ours and all previous studies or platyrrhines used the lower first molar. We speculate that the different findings for the two teeth have to do with the overall biomechanics of the platyrrhine masticatory system. Platyrrhines have a tendency to reduce the size of the molar battery from back to front. Callitrichines have lost the M_3/3_ in most cases but even large bodied 3-molared platyrrhines show an allometric trend toward M_3/3_ reduction that extends mesially to encompass the M_2/2_. Thus, M_2/2_ size is negatively allometric relative to M_1/1_. Bearing this in mind, we used M_1/1_ anticipating future research on smaller-bodied platyrrhines like callithrichines with third molar loss and extreme second molar reduction.

### Body size and diet

Body size has a relatively predictable association with broad diet categories due to metabolic demands of food acquisition and processing [101]. Larger-bodied mammals require less caloric input per gram of body mass than do much smaller mammals. Owing to the absolute quantity of resources required for sustenance, a larger-bodied mammal is unlikely to be able to meet its nutritional demands for protein by eating non-social insects, while a small-bodied mammal (generally, below 500g) may be able to do so. The dataset used herein does not include the smallest bodied and most insectivorous primate species, nevertheless, as expected, we find that molar length on its own goes a long way towards predicting the broad dietary regime of a platyrrhine primate. We found that the addition of body size information (molar length) improves the DFA success rate for assigning individual specimens to diet categories for all variables; however, the classification rates for RFI by itself, in particular, are quite low without associated body size information. This is not surprising given the broad degree of overlap in RFI values among diet categories for both the upper and lower molars. Thus, for our broadly based sample of middle- to large-bodied platyrrhines, we do not find RFI to be particularly useful for assigning species to dietary categories, as they do not provide much information above that demonstrated by molar length alone. We do, however, recommend the consideration of body size as an informative variable in dietary reconstructions.

## Conclusions

1. We explored the ability of dental indices on the lower and upper first molars in medium to large-bodied platyrrhines to differentiate among diet classes. Three diet categories were proposed: frugivore”, “folivore”, and “hard object feeder”.
2. When controlling for sample size and phylogenetic effects, M_1_ SQ most accurately allocates individual specimens and species to the dietary categories “frugivore”, “folivore”, and “hard object feeder”. First molar RFI and OR, are less successful in DFA analyses. We interpret this result as stemming from the fact that RFI and OR incorporate added functional elements of tooth design, such as those that prolong functional lifespan in the face of enamel wear (e.g., crown height), or protect the gingival and reduce the likelihood that teeth will fracture (cingulum development). These added elements might not correspond to the immediate functional aspects of mechanical preparation—breaking up food so as to increase its surface area and speed digestive processes by enzyme and microbial action.
3. Upper first molar indices, particularly SQ, also contain significant dietary information. However, the accuracy of the assignment of individuals or species to diet categories is poorer in general than accuracy achieved by corresponding measures on the lower molars.
4. When both upper and lower indices are considered together with tooth length, we achieve a 93% success rate in allocation of individuals to three-diet category scheme and 92.2% to the four-diet scheme. Species are correctly allocated to diet groups 100% of the time.
5. The results of this study open the way for improved accuracy in inferring the diets of extinct platyrrhines for which both upper and lower molars are available, or, for taxa known only from upper molars. As well, it offers a new window into the dietary behavior of Eocene and Oligocene stem anthropoids and early catarrhines.

## Supporting Information

S1 Table Discriminant Function Analysis results. Sheet 1 for lower molars. Sheet 2 for upper molars. Sheet 3 for combined upper and lower molars.
(XLSX)

S2 Table Measurement data. Data for individual specimens measured in this study.
(XLSX)
